# Structural biology of the LRRK2 GTPase and kinase domains: implications for regulation

**DOI:** 10.3389/fnmol.2014.00032

**Published:** 2014-05-05

**Authors:** Bernd K. Gilsbach, Arjan Kortholt

**Affiliations:** Department of Cell Biochemistry, University of GroningenGroningen, Netherlands

**Keywords:** LRRK2, Roco, structure, kinase, G-protein, Parkinson’s disease

## Abstract

Human leucine rich repeat kinase 2 (LRRK2) belongs to the Roco family of proteins, which are characterized by the presence of a Ras-like G-domain (Roc), a C-terminal of Roc domain (COR), and a kinase domain. Mutations in LRRK2 have been found to be thus far the most frequent cause of late-onset Parkinson’s disease (PD). Several of the pathogenic mutations in LRRK2 result in decreased GTPase activity and enhanced kinase activity, suggesting a possible PD-related gain of abnormal function. Important progress in the structural understanding of LRRK2 has come from our work with related Roco proteins from lower organisms. Atomic structures of Roco proteins from prokaryotes revealed that Roco proteins belong to the GAD class of molecular switches (G proteins activated by nucleotide dependent dimerization). As in LRRK2, PD-analogous mutations in Roco proteins from bacteria decrease the GTPase reaction. Studies with Roco proteins from the model organism *Dictyostelium discoideum* revealed that PD mutants have different effects and most importantly they explained the G2019S-related increased LRRK2 kinase activity. Furthermore, the structure of *Dictyostelium* Roco4 kinase in complex with the LRRK2 inhibitor H1152 showed that Roco4 and other Roco family proteins can be important for the optimization of the current, and identification of new, LRRK2 kinase inhibitors. In this review we highlight the recent progress in structural and biochemical characterization of Roco proteins and discuss its implication for the understanding of the complex regulatory mechanism of LRRK2.

## INTRODUCTION

Parkinson’s disease (PD) affects 1–2% of the population above the age of 65 and is the second most common neurodegenerative disease (Lees et al., 2009). PD causes the loss of dopaminergic neurons in the substantia nigra and is associated with the formation of fibrillar aggregates that are composed of α-synunclein and other proteins. The loss of those neurons leads to the major hallmarks of PD: tremor, bradykinesia, rigidity, and postural instability. Today several genes have been found to be involved in PD, among them the PARK8 locus that encodes for Leucine rich repeat kinase 2 (LRRK2). Mutations in LRRK2 have been found to be the most frequent cause of late onsets PD and are found in both hereditary and sporadic forms of PD (Paisán-Ruíz et al., 2004; Zimprich et al., 2004; Bekris et al., 2010). LRRK2 has been linked to a multitude of cellular functions and pathways, including regulation of neurite outgrowth, Wnt signaling, mitochondrial disease, and autophagy (Dächsel et al., 2010; Winner et al., 2011; Berwick and Harvey, 2012; Papkovskaia et al., 2012). Several studies have identified interaction partners of LRRK2, including 14-3-3, Tubulin, ArfGAP1, Rac1, and DVL (Sancho et al., 2009; Chan et al., 2011; Kawakami et al., 2012; Xiong et al., 2012; Dzamko et al., 2013; Fraser et al., 2013). Despite all this accumulating data, substantial gaps remain in the knowledge about the underlying pathways of LRRK2 mediated PD.

LRRK2 belongs to the Roco superfamily of proteins, which constitutes a novel multi-domain family of Ras-like G-proteins (Bosgraaf and Van Haastert, 2003; Marín et al., 2008). LRRK2 consists of armadillo repeats (ARM), ankyrin repeats (ANK), leucine-rich repeats (LRR), Ras of complex (Roc), C-terminal of Roc (COR), kinase and a WD40 domains (Mills et al., 2014). PD mutations are accumulated around the central core of the protein, two mutations are found in the Roc domain, one in the COR domain, and two in the kinase domain. In addition, two variants have been identified that act as risk factors for sporadic PD, one in the COR domain and one in the WD40 repeats (Cookson, 2010; Cookson and Bandmann, 2010). The multiple disease-linked mutations in LRRK2 represent a unique opportunity to explore the activation mechanism of the protein and its mis-regulation in PD. In this review we will focus on the recent progress in the structural and biochemical characterization of LRRK2 and discuss this in context of the LRRK2 activation mechanism.

## HOMOLOGOUS ROCO PROTEINS AS STRUCTURAL MODEL FOR LRRK2

**FIGURE 1:**
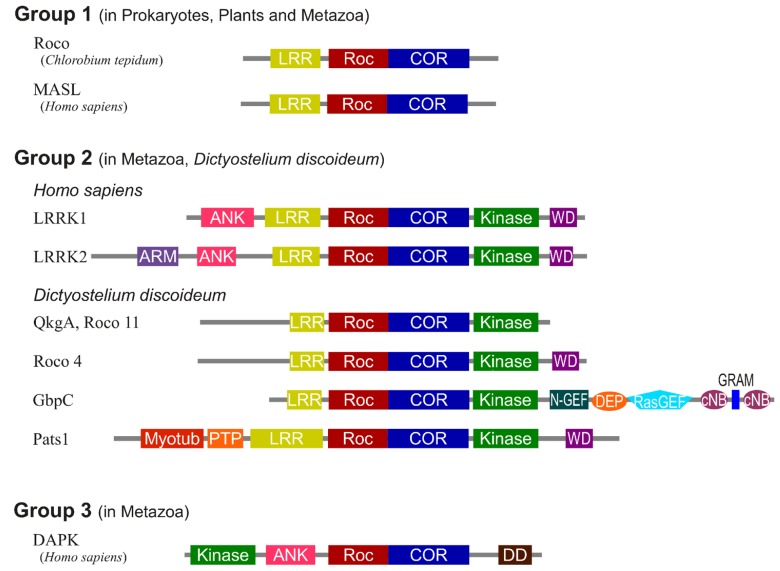
**Domain topology of the Roco family proteins.** The domains are Leucine rich repeats (LRR), Ras of complex proteins (Roc), C-terminal of Roc (COR), Ankyrin repeats (ANK), WD40 repeats (WD), Armadillo repeats (ARM), N-terminal motif of RasGEF (N-GEF), Ras guanine nucleotide exchange factor domain (RasGEF), cyclic nucleotide binding domain (cNB), glucosyltransferases, Rab-like GTPase activators and myotubularins domain (GRAM), N-terminal myotubularin-related domain (myotub), protein tyrosine phosphatase domain (PTP), and death domain (DD).

So far it has been a major challenge to isolate sufficient high-quality recombinant protein of full-length LRRK2 and/or domains thereof. Therefore, important structural understanding has come from work with related Roco proteins from bacteria and *Dictyostelium discoideum* (Gotthardt et al., 2008; Gilsbach et al., 2012). Roco proteins are characterized by the occurrence of a Roc domain, which has high homology to proteins of the Ras superfamily and possesses all five G motifs that are required for guanine nucleotide binding. Roc always forms an inseparable tandem with the COR domain, a 300–400 long stretch of amino acids with no significant homology to other described domains. Roco proteins were first described in *D. discoideum* and have since been identified in prokaryotes, plants and metazoans (Bosgraaf and Van Haastert, 2003; Marín et al., 2008). However, they seem to be absent in yeast and Plasmodium. Four Roco proteins are identified in vertebrates, called LRRK1, LRRK2, death-associated protein kinases-1 (DAPK1), and malignant fibrous histiocytoma amplified sequences with leucine-rich tandem repeats 1 (MASL) (**Figure 1**). Remarkably, the slime mold *D. discoideum* contains 11 Roco family members. Based on domain topology, Roco proteins can be divided into three separate groups (Bosgraaf and Van Haastert, 2003). MASL belongs to the first group of Roco proteins, which is also found in other metazoan, plants and prokaryotes. In these proteins the RocCOR tandem is always preceded by an LRR domain. The human proteins LRRK2 and LRRK1 belong to the second group of Roco proteins. These proteins, which are also present in *D. discoideum* and metazoans, always have in addition to the RocCOR tandem an N-terminal LRR and C-terminal kinase domain. The third group of Roco proteins, which is only found in metazoans, is characterized by the presence of a tumor-suppressor DAPK domain. Besides this general domain composition, individual Roco proteins are found to be combined with a diversity of additional domains such as protein–protein interaction domains, Guanine nucleotide exchange factor (GEF), and Regulator of G-protein Signalling (RGS) domains. Although there is a high variation in these additional regulatory domains among the Roco proteins, as described below, previous studies have shown that the structure, function and regulation of the catalytic core is conserved.

## THE LRRK2 KINASE DOMAIN

The kinase domain of LRRK2 has been extensively studied after its discovery. Kinases transfer the γ-phosphate of ATP to a target protein. Phosphorylation of proteins as a regulatory mechanism was discovered by Krebs and Fisher (1956), in their studies of glycogen phosphorylase. Nowadays, it is known that kinases are essential regulators of almost every signal transduction cascade. Kinases can be classified into three groups, the majority belongs to the group of serine/threonine kinases, a much smaller amount to the class of tyrosine kinases and only a few are classified as atypical kinases (Manning et al., 2002; Taylor and Kornev, 2011; Endicott et al., 2012).

**FIGURE 2:**
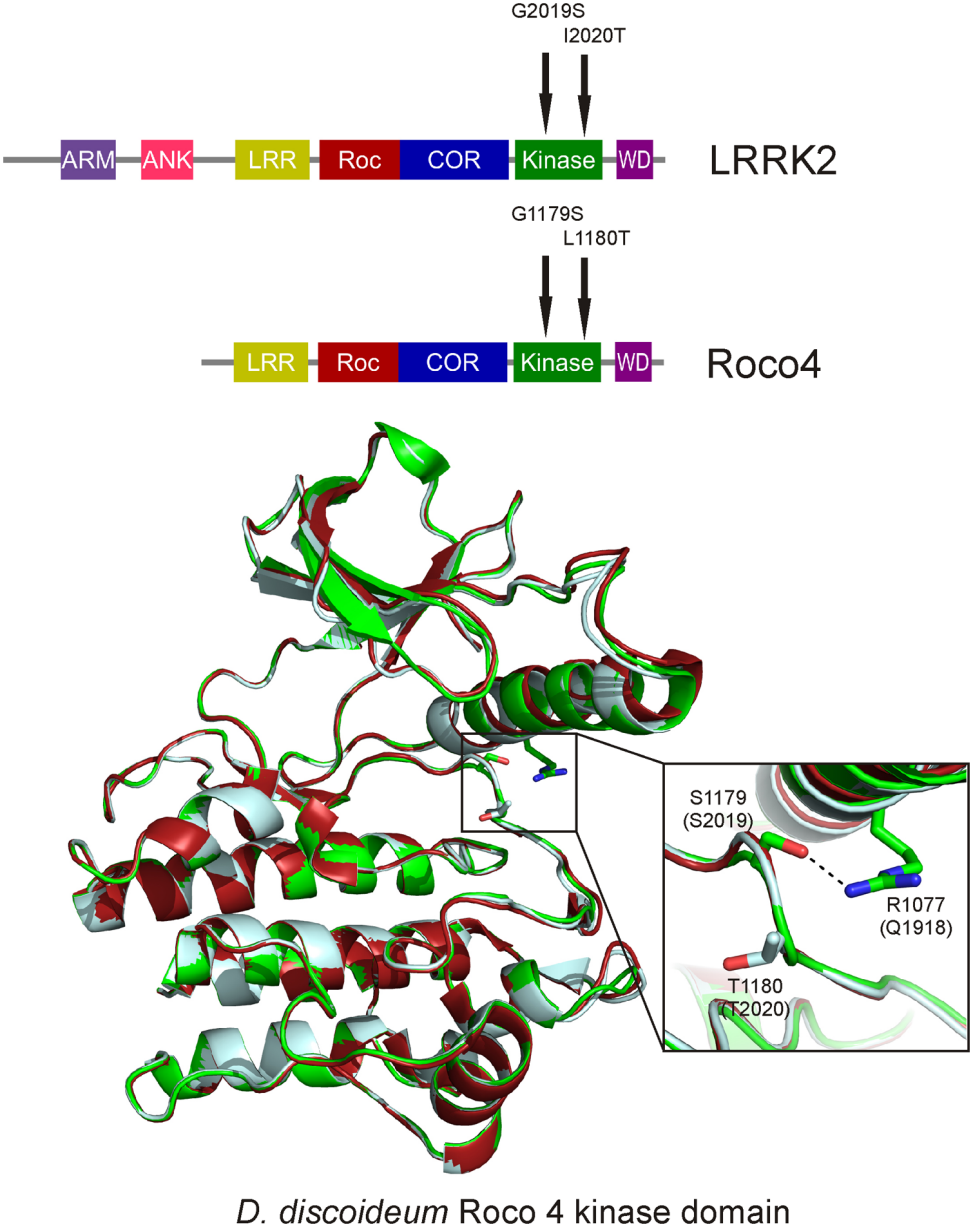
**Overlay of the *Dictyostelium* Roco4 kinase wt, the G1179S (G2019S) and the L1180T (I2020T) structures.** The wt structure is shown in red, the G1179S (G2019S) in green and the L1180T (I2020T) in light blue. Enlarged; PD mutation shown as sticks. The dashed line indicates the stabilizing hydrogen-bond.

LRRK2 and Roco proteins are serine/threonine specific kinases. Our previous solved structure of the kinase domain of* Dictyostelium* Roco4 in its active and inactive state, gave insight into the regulation mechanism of this group of kinases (Gilsbach et al., 2012). *Dictyostelium* Roco4 has the same domain architecture as LRRK2, but is biochemically and structurally more tractable than LRRK2. Like almost all kinases, the Roco4 kinase structure consists of a canonical, two-lobed kinase structure, with an adenine nucleotide bound in the conventional nucleotide-binding pocket (**Figure 2**). The smaller N-terminal lobe is mostly composed of anti-parallel β sheets and contains the conserved αC-helix. The bigger C-terminal lobe mostly consists of α-helices and contains the activation loop with the conserved N-terminal DFG motif. The ATP binding site is formed by a cleft between those lobes and forms the catalytic site of the kinase together with the activation loop and αC-helix. The formation of a polar contact between Roco4 Lys 1055 from the β3-strand and Glu1078 from the αC-helix is essential for correct positioning of the αC-helix. The DFG motif is essential for catalysis: the Asp makes contact with all three ATP phosphates either directly or via coordination of a magnesium ion; the Phe makes hydrophobic contacts to the αC-helix and the HxD motif and is responsible for the correct positioning of the DFG motif. One can distinguish two conformations: a DFG-in (active) and a DFG-out (inactive) conformation. In the structure of active (phosphorylated) Roco4 kinase, the activation loop is visible and ordered. In contrast, the activation loop is not visible and is flexible in the structure of inactive (dephosphorylated) Roco4 kinase (Huse and Kuriyan, 2002; Taylor and Kornev, 2011).

This mechanism to switch from an inactive to an active state is conserved in most kinases and often involves autophosphorylation of one or more residues in the activation loop. Autophosphorylation not only results in the reorientation of the activation loop, but often also alters ATP binding and/or interaction with substrates (Huse and Kuriyan, 2002; Kornev et al., 2006). Autophosphorylation of LRRK2 and related Roco proteins was shown by various studies (Lobbestael et al., 2012; Zhao et al., 2012). LRRK2 possesses three potential phosphorylation sites in the activation loop (Thr2031, Ser2032, and Thr2035), while four putative phosphorylation sites (Ser1181, Ser1184, Ser1187, and Ser1189) are present in the same region of Roco4. *In vitro* and *in vivo* analysis revealed that in both LRRK2 and Roco4, only the two latter phosphorylation sites are important for function *in vivo* (Li et al., 2010; Gilsbach et al., 2012). In addition, several LRRK2 autophosphorylation sites outside the activation loop have been identified, most of which are located in the Roc domain. Importantly, mutation of several of these residues, or inhibition of kinase activity with inhibitors, completely rescues neurite outgrowth in LRRK2 PD mutant strains (MacLeod et al., 2006; Herzig et al., 2011; Yao et al., 2013). This suggests that LRRK2 kinase-mediated phosphorylation events are important for both the intramolecular activation mechanism as well for downstream signaling.

## MECHANISM OF PD-MUTATION IN THE LRRK2 KINASE DOMAIN

It has been shown that kinase activity is essential for LRRK2-induced neuronal toxicity (Greggio et al., 2006; Smith et al., 2006). However, conflicting data regarding kinase activity of PD-related mutants have been published. Increased kinase activity only has been consistently shown for the most prevalent LRRK2 Gly2019Ser mutation, whereas for the other mutations, either no effect or even a decreased kinase activity has been reported (Reviewed by Greggio and Cookson, 2009). LRRK2 Gly2019 is located within the conserved DFG motif and critically linked to a 2- to 4-fold increase in kinase activity (West et al., 2005; Greggio et al., 2006; Jaleel et al., 2007; Anand et al., 2009). The molecular mechanism by which this mutation enhances the catalytic activity of LRRK2 was resolved by our study with *Dictyostelium* Roco4 as a model (Gilsbach et al., 2012). LRRK2 Gly2019 corresponds to Roco4 Gly1179. As expected, introducing the PD mutation at this position leads to increased kinase activity. The Roco4 Gly1179Ser mutation does not result in large changes in the overall structure, however, closer observation reveals that Ser1179 makes a new hydrogen bond with Arg1077, thereby presumably stabilizing the activation loop and the αC-helix in their active conformation (**Figure 2**). Roco4 Arg1077 is conserved in almost all Roco proteins and corresponds to LRRK2 Gln1918. Consistent with the proposed mechanism: the Roco4 double mutant Gly1179Ser/Arg1077Ala and the homologous LRRK2 double mutant Gly2019Ser/Gln1918Ala, in which the new hydrogen bond is no longer possible, have again normal wild-type kinase activity (Gilsbach et al., 2012).

Both the LRRK2 Ile2020Thr PD mutant and the corresponding Roco4 Leu1180Thr mutant have a slightly decreased kinase activity (Jaleel et al., 2007; Gilsbach et al., 2012). The structure of Roco4 Leu1180Thr does not directly explain this decreased kinase activity: the Thr1180 side-chain points into the solvent and most likely does not directly interfere with active site configuration. It has been speculated that the higher neurotoxicity of this mutant might be due to a higher susceptibility of the mutant to intracellular degradation (Smith et al., 2006; Ohta et al., 2010). Others postulated that in analogy to what has been shown for B-RAF mutations, LRRK2 works in tandem such that the interaction between wild-type and LRRK2-Thr2020 might increase kinase activity (Wan et al., 2004). Alternatively, the Ile2020Thr could affect intramolecular interactions with other domains and thereby indirectly influence kinase activity. Importantly, the Roco4 structures show that the PD-related effect of LRRK2 mutations result from different defects in the LRRK2 activation mechanism.

## STRUCTURAL-BASED OPTIMIZATION OF KINASE INHIBITORS

Kinases are one of the most potent classes of drug targets and have been effectively used in the treatment of cancer, and for immunological, neurological and infectious diseases (Cohen, 2002). Several kinase inhibitors have been identified that are selective for LRRK2 and brain penetrant (Deng et al., 2011; Ramsden et al., 2011; Choi et al., 2012; Reith et al., 2012; Zhang et al., 2012). However, long-term inhibition of LRRK2 by many of these inhibitors leads to kidney abnormality, similar to what has been observed in LRRK2 knock-out mice (Herzig et al., 2011; Tong et al., 2012; Ness et al., 2013). Most likely, the ATP binding pocket is the direct target of many of these inhibitors, but the exact binding mechanism is unknown. Previously, the structure of the *Dictyostelium* Roco4 kinase in complex with the LRRK2 inhibitor H1152 was solved (Gilsbach et al., 2012). This shows that Roco4 can be used as an important tool to biochemically and structurally characterize LRRK2 inhibitor binding in more detail (Gilsbach et al., 2012). Furthermore, Roco4 structures will allow the building of a reliable model of LRRK2 for computer-aided drug development, while the biochemical tractability of Roco4 allows the *in vitro* screening of inhibitor libraries.

## STRUCTURAL CHARACTERIZATION OF THE Roc-COR TANDEM

So far, the function of the Roc domain of LRRK2 is not completely understood. However, it has been shown that the G-domain of LRRK2 functions as a bona fide GTP-binding protein and that GTP binding is essential for the regulation of kinase activity (Taymans, 2012; Biosa et al., 2013). Harvey and Kirsten already showed the oncogenic effect of mutated Ras in the 1960s, and since the function of small G-proteins has been extensively studied (Vetter and Wittinghofer, 2001; Bos et al., 2007; Csépányi-Kömi et al., 2012). G-proteins switch between an active GTP- and inactive GDP-bound state. G domains, including Roco proteins, contain the five highly conserved motifs, G1–G5, which are responsible for nucleotide binding. The G1 motif, also called p-loop, is essential for the binding of the α- and β-phosphate of the nucleotide, as well as for the interaction with a magnesium-ion in the nucleotide binding pocket. G-domains have a universal switch mechanism that carries out the basic function of nucleotide binding and hydrolysis (Vetter and Wittinghofer, 2001). The structures of the GDP- and GTP- bound state of Ras only differ in the switch regions, which are in an active and inactive conformation, respectively (Vetter and Wittinghofer, 2001). Despite this small conformational change, only GTP-bound G-protein has a high affinity for effector proteins.

**FIGURE 3:**
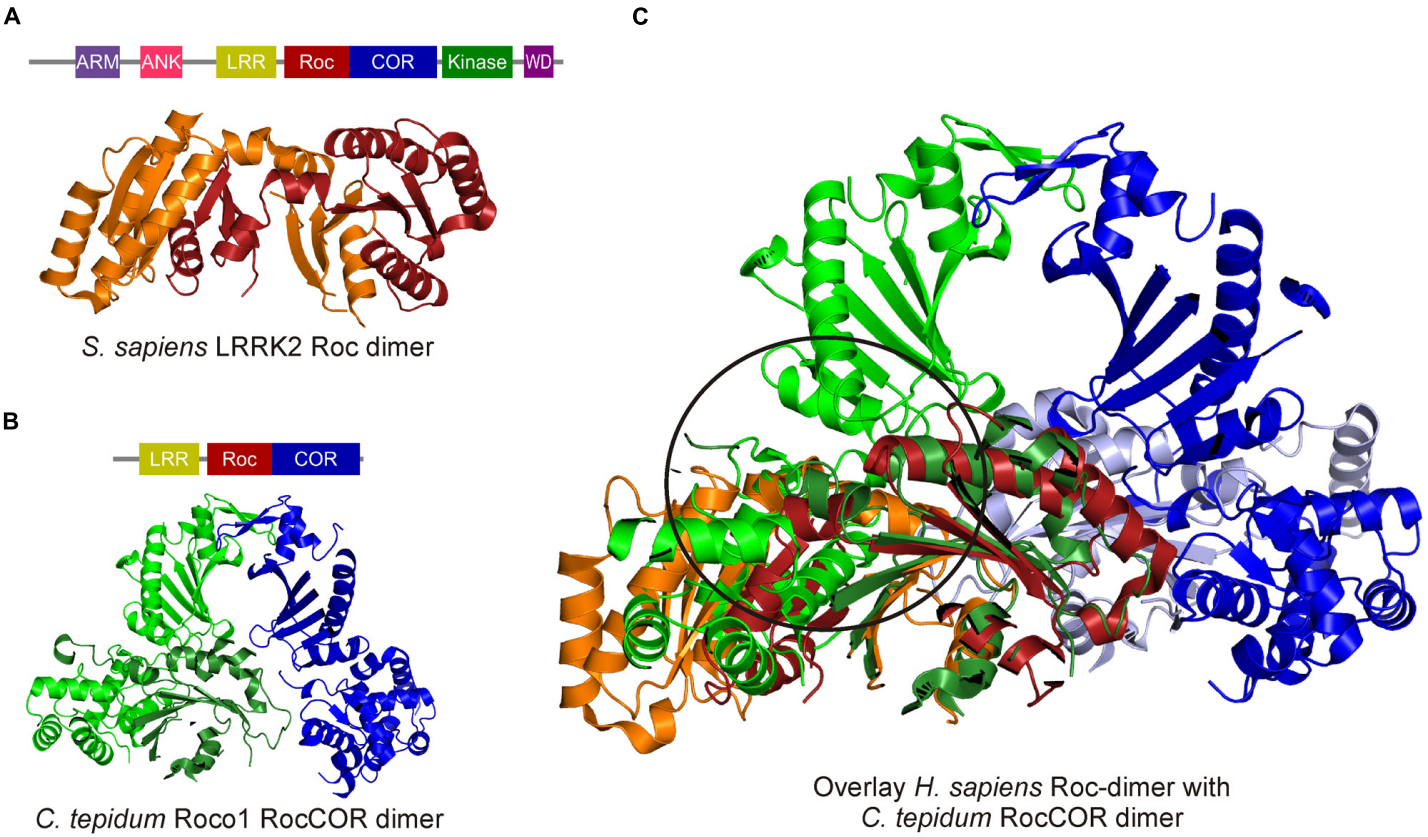
**Crystal structures of the human swapped Roc dimer and the *C. tepidum* RocCOR dimer. (A)** Human Roc Dimer depicted as a cartoon with Roc-A in orange and Roc-B in red. Above the domain representation of LRRK2 is shown. **(B)** Domain representation of *C. tepidum* and below a cartoon representation of the *C. tepidum* RocCOR structure with RocCOR-A in green and COR-B in blue. **(C)** Overlay of the two structures Roc-A (orange) of the human protein clashes with the N-terminal part of the *C. tepidum* COR-A (green). [PDB: 2ZEJ (human Roc), 3DPU (C. tepidum RocCOR)].

In Roco family members, the G-domain always occurs in tandem with the COR domain. There are two crystal structures comprising the Roc G-domain available: one structure of the LRRK2 Roc domain and one of the Roc-COR tandem of the Roco protein from *Chlorobium tepidum* (Deng et al., 2008; Gotthardt et al., 2008). Surprisingly, the structure of the LRRK2 Roc domain revealed a swapped dimer: in which the N-terminal part of one G-domain interacts with the C-terminal of the other, thereby forming a constitutive dimer (Deng et al., 2008). In contrast, the Roc domain in the *C. tepidum* RocCOR dimer structure shows the typical small G protein fold with six β-strands and helices on both sides and an additional N-terminal helix, termed α_0_-helix (**Figure 3**). The COR domain consists of two parts: the highly conserved N-terminal part interacts with the Roc domain and the less conserved C-terminal part functions as dimerization device. It seems rather unlikely that the human RocCOR tandem has a different folding than that of the bacterial Roco protein. Importantly, an overlay of the human Roc and the bacterial RocCOR structure revealed major clashes of the highly conserved N-terminal part of the COR domain with the swapped Roc dimer [**Figure 3**, (Gotthardt et al., 2008)]. Furthermore, Deng et al. (2008) could not convincingly show dimer formation of the Roc domain in solution, while Liao et al. (2014) showed that human Roc forms primarily a monomer in solution with low GTPase activity. (Deng et al., 2008; Liao et al., 2014) Together, this strongly suggest that, like all previously observed swapped G-protein structures (Chavas et al., 2007), the LRRK2 Roc structure is a crystallographic artifact.

## REGULATION OF THE G-DOMAIN AND EFFECT OF PD-MUTATIONS

The switch between the active and inactive state of small G proteins is dependent on regulatory proteins. Small G-proteins have a very high nucleotide affinity (nM–pM range), GEFs reduce this affinity by many orders and thereby promote nucleotide release. This subsequently facilitates binding of GTP, which is present in about 30-fold excess over GDP in the cytosol of the cell (Bernards and Settleman, 2004). The intrinsic GTPase activity of small G-proteins is extremely low; therefore GTPase activating proteins (GAPs), which increase the intrinsic GTPase activity by a thousand fold or more, are necessary to switch the protein off (Scheffzek, 1997; Bernards and Settleman, 2004).

There are a few reports describing GAPs and GEFs for LRRK2. Surprisingly none of these putative regulators directly bind to the Roc domain (Stafa et al., 2012; Xiong et al., 2012; Häbig et al., 2013). LRRK2 and all Roco proteins studied so far have a much lower nucleotide affinity (μM range) compared to other small G-proteins and are therefore not strictly dependent on GEFs for activation (Ito et al., 2007; Gotthardt et al., 2008; Liao et al., 2014). However, in some transient responses, as previously shown for *Dicyostelium* Roco1, additional stimulation of the already high intrinsic nucleotide exchange rate by GEFs might be required. It is well known that LRRK2 and other Roco proteins are active as a dimer (Greggio et al., 2008; Berger et al., 2010).

The *C. tepidum* RocCOR structure showed that COR is the dimerization device and that Roco proteins that are not able to dimerize, are subsequently also not able to hydrolyze GTP (Gotthardt et al., 2008). This suggest that Roco proteins belong to the GAD class of molecular switches (G proteins activated by nucleotide dependent dimerization; Gotthardt et al., 2008). Important proteins such as signal recognition particle, dynamin and septins also belong to this class of G-proteins (Gasper et al., 2009). GADs possess usually low nucleotide affinity and the stimulation of the low GTPase activity is completely dependent on dimerization. Consistently, the hydrolysis rate of the monomeric LRRK2 Roc domain is more than 700-fold slower than that of dimeric full-length LRRK2 (Liu et al., 2010; Liao et al., 2014). In GADs, stimulation of GTPase activity is accomplished by nucleotide dependent dimerization; within the complex, one monomer completes the catalytic machinery of the other monomer (Gasper et al., 2009). *C. tepidum* Roco uses, like classical Ras-GAPs, an Arginine finger that is essential for stimulating GTP hydrolysis in the neighboring Roc domain (Gotthardt et al., 2008).

Two common PD-related mutations have been found in the RocCOR domain: Arg1441 with multiple substitutions (Cys/Gly/His) in the Roc domain and Tyr1699Cys in the COR domain (Zimprich et al., 2004). Due to the lack of stable purified recombinant protein it has been so far a challenge to study if these mutations affect the GTPase activity of LRRK2. However, recent data strongly suggest that both the PD mutations in the Roc and the COR result in decreased GTPase activity (Lewis et al., 2007; Li et al., 2007). Importantly, the structure of the *C. tepidum* Roco protein showed that the PD-analogous mutations of the Roc and COR domains are in close proximity to each other at the dimer interface and most likely alter the interaction in the dimer between the Roc and COR domains (Gotthardt et al., 2008). Furthermore, these mutations are present in a region of the protein that is strongly conserved between bacteria and man. Subsequently, the Arg1441Cys and Tyr1699Cys PD mutations, as well as the PD-analogous mutations in the *C. tepidum* protein, do not affect nucleotide binding, but significantly decrease GTPase activity (Guo et al., 2007).

## STRUCTURE OF THE N- AND C-TERMINUS OF LRRK2

In addition to the central core of the protein, Roco proteins contain a large variety of additional C- and N-terminal domains. The N- terminal part of LRRK2 consists of ARM, ANK, and LRR, while a WD40 domain is present at the C-terminus of LRRK2 (Cardona et al., 2014). All these domains are commonly found in signaling proteins, in which they have often a role in protein–protein interaction. Although there are no structures of the N- or C-terminal LRRK2 domains available, these protein–protein domains have a highly conserved fold (Cardona et al., 2014). ARM repeats are about a 42 amino acid long tandem repeat that form a super-helical bundle (Tewari et al., 2010). One mutation within the ARM domain (Glu334Lys) is associated with PD. *In silico* modeling predicts that this mutation changes the electrostatic surface of the domain (Cardona et al., 2014). ANK contains seven repetitive motifs which form helix-loop-helix structures that end in a loop or hairpin (Mosavi and Cammett, 2004). Modeling of the PD-related Pro755Leu and Arg793Met mutants predict that these mutations would affect protein stability or the electrostatic surface, respectively (Cardona et al., 2014). LRRs are made of an 11 amino acid long conserved motif LxxLxLxxNxL (Leucines can be replaced by isoleucine, valine or phenylalanine). These repeats form a parallel β-sheet and end with a α-helix. Multiple LRR repeats together form a characteristic horseshoe like structure (Bella et al., 2008). The recombinant purified LRR domain of LRRK2 is monomeric in solution and PD variants in the LRRs do not alter the overall folding of the protein, suggesting that most likely these mutation affect inter- and intramolecular interactions (Vancraenenbroeck et al., 2012). The LRR domains of LRRK2 and *Dictyostelium* Roco4 are essential for function *in vivo*, but are not required for kinase activity *in vitro* (Iaccarino et al., 2007; van Egmond and van Haastert, 2010). This suggests that the LRRs most likely determine the specificity of the protein by interacting with an upstream activator or downstream target of the protein. Previously, it was shown that the N-terminus of LRRK2 binds in a phosphorylation-dependent manner to 14-3-3 proteins (Nichols et al., 2010). Inhibiting phosphorylation of two LRRK2 residues, Ser910 and Ser935, disrupts binding to 14-3-3 and subsequently leads to strong defects in LRRK2 signaling; the protein is delocalized and accumulates in inclusion-like bodies instead of being transported to the cell membrane (Dzamko et al., 2010).

WD40 domains form a seven-blade propeller-like structure. Each propeller blade is made of four antiparallel β-strands (Xu and Min, 2011). WD40 domains usually have a highly hydrophilic surface and are often involved in membrane binding. Disruption of the LRRK2 WD40 domain results in abolished dimer formation, impaired kinase activity and aberrant protein localization (Jorgensen et al., 2009). Furthermore, the Gly2385Arg PD risk factor mutation causes a decrease in kinase activity and loss of 14-3-3 binding to the N-terminus (Rudenko et al., 2012). Together these results suggest an important role for the WD40 domain in the intramolecular regulation of LRRK2 activity.

## LRRK2 ACTIVATION MODEL

**FIGURE 4:**
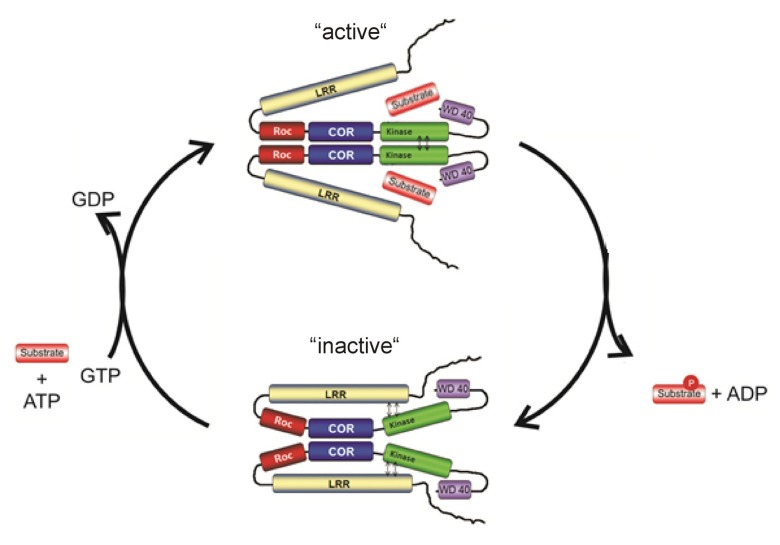
**Working model for the complex LRRK2 activation mechanism**.

Altogether, the structural and biochemical data suggest that LRRK2 activity is regulated by at least two different mechanisms: intramolecular activation and binding of input/substrate to the N- and C- terminal domains (**Figure 4**). The COR domain functions as a dimerization device. Within the dimer, the Roco G-domains are flexible in the GDP-bound inactive state, but in the active form the G-domains come in a more fixed state in close proximity to each other. This conformational change is transmitted to other parts of the protein, which subsequently allows the activation loops of the two kinase protomers to be autophosphorylated and activated. The GTPase reaction is also critically dependent on dimerization, because efficient catalytic machinery is formed by complementation of the active site of one protomer with that of the other protomer. In this way the intramolecular GTPase reaction functions as a timing device for the activation and biological function of Roco proteins. The N- and C-terminal segments of LRRK2 regulate this intramolecular signaling cascade and are important for kinase activity, oligomerization, and/or localization. Most likely the N- and C-terminal protein-protein interaction domains are directly binding upstream proteins and/or downstream effector proteins and thereby determine the specificity of the Roco proteins.

## CONCLUSION

The multiple allosteric and enzymatic functions within one protein make LRRK2 an excellent therapeutic target. So far the major focus has been to develop kinase domain inhibitors as potential PD therapeutics. However, most of the specific LRRK2 inhibitors lead to kidney and lung abnormality. Furthermore, an increased kinase activity has only been thus far reported for G2019S. All other pathogenic mutations show inconsistent, modest or no effect on kinase activity. Importantly, the Roco structures show that PD-mutations have different defects in the LRRK2 activation mechanism. Therefore, alternative approaches that target other domains of LRRK2, including LRRK2, localization, dimerization, or allosteric modulation of the kinase domain may have significantly improved therapeutic benefits. To fully explore these potential targets more knowledge about the complex intramolecular activation mechanism of LRRK2, upstream and downstream regulators, and the cellular function of LRRK2 is needed. A high-resolution structural map of LRRK2 and related Roco proteins is essential in this enterprise.

## Conflict of Interest Statement

The authors declare that the research was conducted in the absence of any commercial or financial relationships that could be construed as a potential conflict of interest.
